# A Portable Smartphone-Based Laryngoscope System for High-Speed Vocal Cord Imaging of Patients With Throat Disorders: Instrument Validation Study

**DOI:** 10.2196/25816

**Published:** 2021-06-18

**Authors:** Youngkyu Kim, Jeongmin Oh, Seung-Ho Choi, Ahra Jung, June-Goo Lee, Yoon Se Lee, Jun Ki Kim

**Affiliations:** 1 Biomedical Engineering Research Center Asan Institute for Life Sciences Asan Medical Center Seoul Republic of Korea; 2 Department of Convergence Medicine College of Medicine University of Ulsan Seoul Republic of Korea; 3 Department of Otorhinolaryngology-Head and Neck Surgery Asan Medical Center Seoul Republic of Korea; 4 Department of Otorhinolaryngology-Head and Neck Surgery Eulji Medical Center Eulji University School of Medicine Seoul Republic of Korea

**Keywords:** smartphone, mobile phone, endoscope, high-speed imaging, vocal cord, low-cost device, mHealth, otorhinolaryngology, head and neck, throat

## Abstract

**Background:**

Currently, high-speed digital imaging (HSDI), especially endoscopic HSDI, is routinely used for the diagnosis of vocal cord disorders. However, endoscopic HSDI devices are usually large and costly, which limits access to patients in underdeveloped countries and in regions with inadequate medical infrastructure. Modern smartphones have sufficient functionality to process the complex calculations that are required for processing high-resolution images and videos with a high frame rate. Recently, several attempts have been made to integrate medical endoscopes with smartphones to make them more accessible to people in underdeveloped countries.

**Objective:**

This study aims to develop a smartphone adaptor for endoscopes, which enables smartphone-based vocal cord imaging, to demonstrate the feasibility of performing high-speed vocal cord imaging via the high-speed imaging functions of a high-performance smartphone camera, and to determine the acceptability of the smartphone-based high-speed vocal cord imaging system for clinical applications in developing countries.

**Methods:**

A customized smartphone adaptor optical relay was designed for clinical endoscopy using selective laser melting–based 3D printing. A standard laryngoscope was attached to the smartphone adaptor to acquire high-speed vocal cord endoscopic images. Only existing basic functions of the smartphone camera were used for HSDI of the vocal cords. Extracted still frames were observed for qualitative glottal volume and shape. For image processing, segmented glottal and vocal cord areas were calculated from whole HSDI frames to characterize the amplitude of the vibrations on each side of the glottis, including the frequency, edge length, glottal areas, base cord, and lateral phase differences over the acquisition time. The device was incorporated into a preclinical videokymography diagnosis routine to compare functionality.

**Results:**

Smartphone-based HSDI with the smartphone-endoscope adaptor could achieve 940 frames per second and a resolution of 1280 by 720 frames, which corresponds to the detection of 3 to 8 frames per vocal cycle at double the spatial resolution of existing devices. The device was used to image the vocal cords of 4 volunteers: 1 healthy individual and 3 patients with vocal cord paralysis, chronic laryngitis, or vocal cord polyps. The resultant image stacks were sufficient for most diagnostic purposes. The cost of the device including the smartphone was lower than that of existing HSDI devices. The image processing and analytics demonstrated the successful calculation of relevant diagnostic variables from the acquired images. Patients with vocal pathologies were easily differentiable in the quantitative data.

**Conclusions:**

A smartphone-based HSDI endoscope system can function as a point-of-care clinical diagnostic device. The resulting analysis is of higher quality than that accessible by videostroboscopy and promises comparable quality and greater accessibility than HSDI. In particular, this system is suitable for use as an accessible diagnostic tool in underdeveloped areas with inadequate medical service infrastructure.

## Introduction

The endoscope enables optical visualization of internal organs and is a fundamental diagnostic device in clinical fields; unique properties of endoscopic systems are indispensable in diagnostics [1], biopsy [2], and minimally invasive surgery [3]. To acquire more accurate and specific diagnoses, endoscope systems have been developed with higher resolution [4], greater imaging speed [5,6], quantitative image analysis [7,8], and higher performance. With these advantages, endoscopy is now considered to be an essential procedure for a variety of clinical applications, including clinical voice evaluation.

Imaging of vocal cord vibrations is one of many techniques used for clinical voice assessment. It is commonly accepted that vocal cord vibration irregularities strongly correlate with voice disorders. However, since the frequencies of vocal cord vibrations are approximately 80-240 Hz, standard videostroboscopy at 60 frames per second (fps) does not permit the capture of vocal cord movement aside from stable and periodic states [9-11]. Alternative methods have been studied to overcome the limitations of videostroboscopy for imaging vocal cord vibrations. The most promising approach is high-speed digital imaging (HSDI) [12], which typically captures images between 4000 and 8000 fps [13]. The high frame rate of HSDI allows for the capture of vocal vibrations from 80-240 Hz, which cannot be observed in standard 60 fps imaging systems. Furthermore, HSDI provides a sufficient resolution of more than 256 × 256 pixels [14]. Although the resolution is hardly comparable to that of HD (high definition), it is sufficient for medical analysis. Use of HSDI for clinical voice evaluation has been extensively demonstrated [10,15,16]. However, despite several advantages, HSDI is not widely used in clinical diagnosis because systems with better performance than laryngostroboscopy are generally expensive, require specialized technology, and have large data footprints [13].

Endoscopic diagnosis is mostly inaccessible for patients in underdeveloped and developing countries. Indeed, even in the most developed and well-equipped hospitals in Nigeria, there may be only one functional gastroscope and/or colonoscope, for which the accessories are often deficient. Furthermore, the majority of the teaching hospitals in Nigeria have no facilities for therapeutic endoscopy [17]; this leads to a lack of experience in endoscopy for medical students, which can lead to unsatisfactory or even fatal endoscopy procedures. Furthermore, more serious issues, including inadequate maintenance, cross-infections, and insufficient performance of devices, have also been found in developing countries. In facilities with insufficient endoscopic gastroscope and colonoscope accessories, HSDI devices are difficult to furnish because of their high cost and additional technology requirements. This seriously restricts clinical voice evaluation in underdeveloped countries since HSDI has become an important part in the diagnosis of voice disorders. As with many problems regarding endoscopy in developing countries, affordable and more manageable endoscopic HSDI systems are urgently required.

Several point-of-care diagnostic devices have been invented in response to the demand in developing and underdeveloped countries. Prior to the age of integrated electronics, handheld point-of-care devices were largely restricted to diagnostic strips, which relied heavily on color changes due to chemical reactions [18]. With the ubiquity of smartphones, the rate of invention of electronic point-of-care devices has drastically increased. Modern smartphones offer a computing performance comparable to that of personal computers, and imaging quality similar to that of professional camera devices. Consequently, there have been many attempts to use smartphones for point-of-care diagnosis. For example, smartphone cameras can be used to distinguish reaction intensities, and several smartphone attachments have been introduced for biological analysis similar to that performed by enzyme-linked immunosorbent assay (ELISA) [19], fecal hemoglobin detection [20], and electrochemical monitoring of blood contents [21]. This represents an evolution of traditional point-of-care strip diagnosis, in which a smartphone can be used to distinguish the intensity of a biological reaction. Other proposed applications of the smartphone camera for point-of-care diagnosis include clinical microscopy, endoscopic imaging, video nasolaryngoscopy, flexible robotic endoscopy, etc [22-28].

In this study, we introduce a smartphone-based endoscopic imaging device and validate its imaging performance relative to videostroboscopy and HSDI. The smartphone adaptor was designed and manufactured using 3D design software and selective laser melting (SLM)–based 3D printing. This customized smartphone adaptor for the endoscope has minimal and low-cost optical components for easy maintenance and control. Unlike previous studies that only used smartphones for acquisition of regular images and videos, in this study, the specialized high-speed imaging function of the smartphone was applied for HSDI of vocal cord vibrations. With this functionality, high-speed vocal cord videos were acquired at 940 fps following standard clinical protocols and analyzed using postprocessed imaging techniques such as segmentation and registration to simplify diagnosis. The resultant images are quantitatively more revealing than videostroboscopy, and approach the quality of HSDI due to their high resolution and frame rate. In preclinical image analysis, several diagnostic variables were determined likely to assist in the diagnosis of common vocal cord pathologies. This custom simple, low-cost adaptor promises to reduce the cost of high-speed clinical imaging by an order of magnitude when it can be used as a substitute for standard HSDI. The device demonstrates the promise and emerging capabilities of clinical diagnostic tools that incorporate commodity smartphone technology to bring point-of-care diagnosis to remote locations.

## Methods

### Development of a Smartphone-Adapted Endoscope

**Figure 1:**
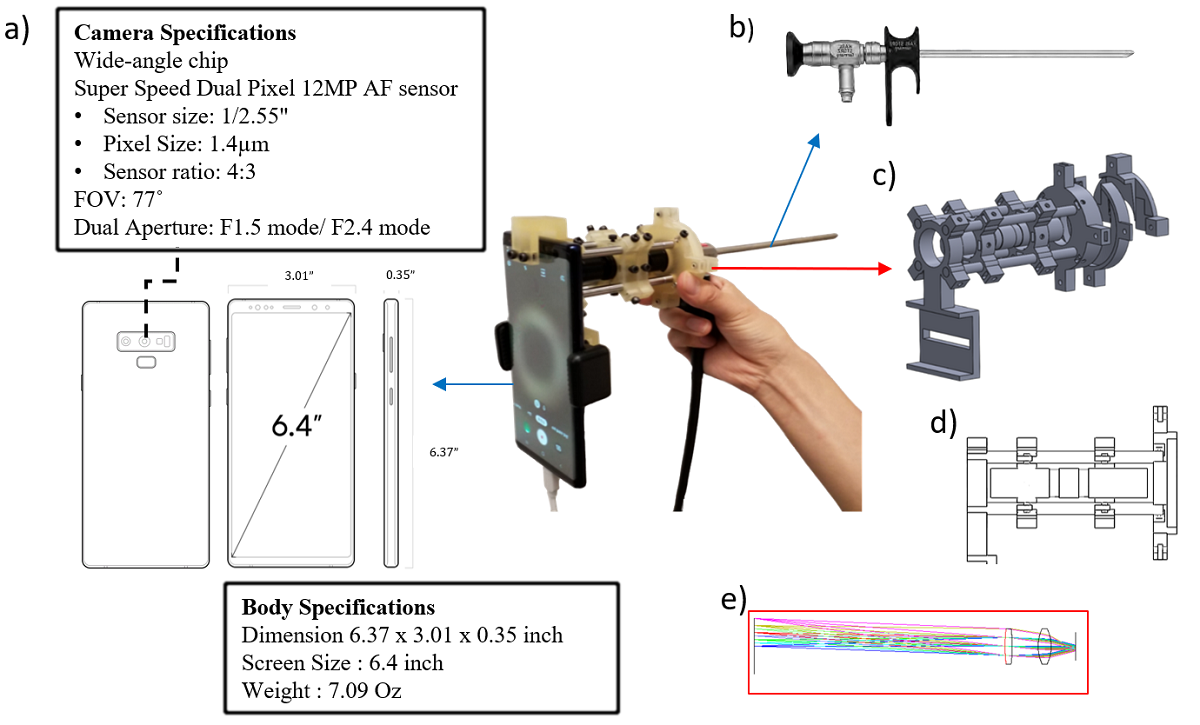
Schematics of a customized smartphone-endoscope adaptor that enables high-speed laryngoscopy. (A) Smartphone camera and body specifications. (B) A photograph of the demonstrated smartphone adaptor incorporating a rigid clinical endoscope. (C) 3D models of the customized smartphone-endoscope adaptor. (D) Cross-sectional view of the customized smartphone-endoscope adaptor. (E) Schematic and lens simulations of the magnification lens system. AF: autofocus, FOV: field of view.

A smartphone adaptor was developed for use in clinical endoscopy (Figure 1) with a standard 70° rigid laryngoscope (Karl Storz Co). The adaptor consists of a smartphone holder, lenses, a lens tube, and mounts for the lens tube and for connection to the endoscope. The holders and connectors were designed using 3D modeling software (Solid Works) and printed by an SLM 3D printer (Objet260, Stratasys Ltd). A lens system was used to magnify the endoscope probe eyepiece image between the endoscope eyepiece and the smartphone camera and was held by 3D-printed parts. To make the system more cost-effective, a combination of 2 biconvex lenses was used instead of expensive achromatic lenses for magnification of the endoscopic image. The focal lengths of the 2 biconvex lenses were 50 mm and 15 mm, respectively; this optical setup could acquire images with approximately 1.5× magnification. Illumination was coupled into the illumination port on the endoscope. For endoscopic illumination, we used a broadband (360-770 nm) LED light source (X-Cite XYLIS, Excelitas Technologies Corp), which was connected to the illumination port of the endoscope with a liquid light guide.

A specific smartphone model (Galaxy Note 9, Samsung) was used to capture endoscopic images at a high frame rate. The catalog specification provided by the manufacturer of the smartphone claims that this specific smartphone model can acquire high-speed images at 960 fps, for a maximum duration of 0.4 seconds, at 1280 × 720 pixel resolution [29]. The complementary metal-oxide-semiconductor sensor of the smartphone is specified for 12.0 megapixels (MP) and 1/2.55 inches (1.4 μm pixel size), and is packaged with a dual aperture mode.

### Statistical and Data Analysis

The acquired high-speed vocal cord vibration images were segmented using the seeded region growing algorithm [30] to determine the amplitude of the vibrations on each side of the glottis [31]. Image analysis software was developed in MATLAB (MathWorks) to calculate the diagnostic parameters, including the vocal cord vibration frequencies, glottal edge phase shifts, and total glottal area changes.

### Clinical Experiment

**Figure 2:**
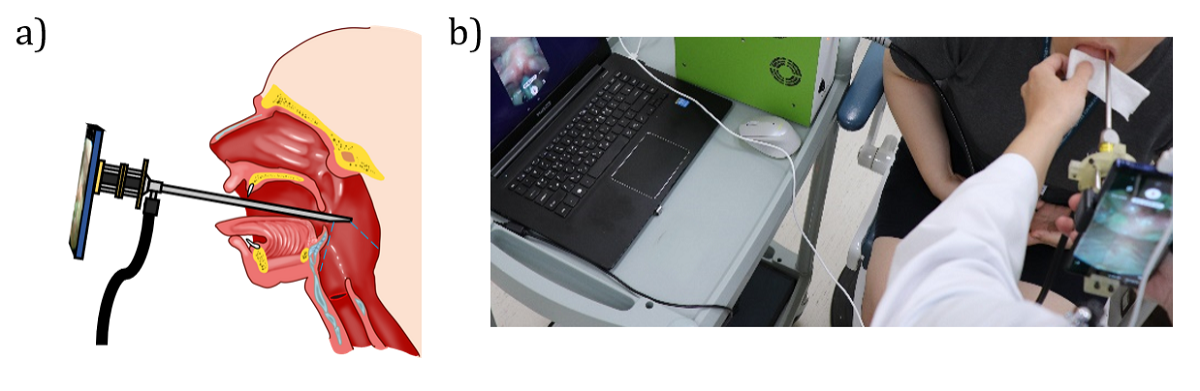
(A) A schematic representation of laryngeal imaging performed by the smartphone-based high-speed imaging system. (B) A clinical demonstration of the smartphone-based high-speed imaging system.

The developed smartphone-based imaging system was used to acquire a high-speed video of the vocal cords of 4 human subjects—1 healthy individual and 3 patients with known vocal pathologies. A board-certified otolaryngologist at Asan Medical Center with 15 years of experience followed common videokymography routines [11] to capture high-speed diagnostic videos in a clinical environment (Figure 2). The routine vocal cord examination was as follows: the first step was to interview the patient and assess their symptoms and voice status, and the second step was videostrobolaryngoscopy or videokymography with an endoscope. Depending on the patient’s condition, additional tests such as computed tomography (CT) scans or a blood test may be required.

Before imaging human subjects, the smartphone-based HSDI system was adapted to the clinical endoscope by adjusting the fine focus and fixing the endoscope in place. Following confirmation that the received images were clear and focused, the clinician performed routine vocal cord evaluation using the endoscope-smartphone device. When the diagnosis routine was completed, the endoscope probe was wiped with a disinfectant to avoid cross-contamination between human subjects [32]. The whole procedure took less than a minute, thus avoiding undue burden on volunteers.

The study was approved by the Institutional Review Board (IRB #2020-0798) of Asan Medical Center, Seoul, under the Korean Bioethics and Safety Act and the Korean Medical Device Act.

## Results

### System Characterization

**Figure 3:**
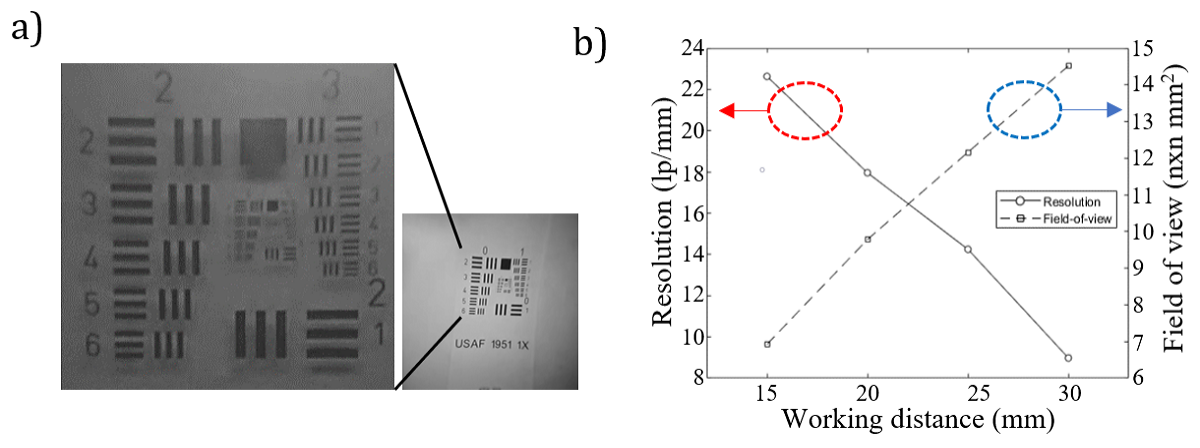
(A) US Air Force target test image taken at 960 fps. (B) A graph of the measured image resolution and field of view as functions of working distance.

The US Air Force resolution target was used to evaluate the imaging performance of the smartphone-endoscope system (Figure 3A). The distance between the end of the endoscope probe and the resolution target was adjusted from 15 mm to 30 mm in order to determine the proper optical field of view (FOV) and resolution. At the highest resolution, the system resolved 23 line pairs per millimeter (lp/mm), which corresponded to 43.478 μm at a working distance of 15 mm. The resolution of the system was observed to decrease as the working distance was increased. In addition, the optical FOV was 7.8 mm × 7.8 mm at a 15 mm working distance, and 14.6 mm × 14.6 mm at a 30 mm working distance; thus the optical FOV increased approximately linearly with the working distance (Figure 3B).

**Figure 4:**
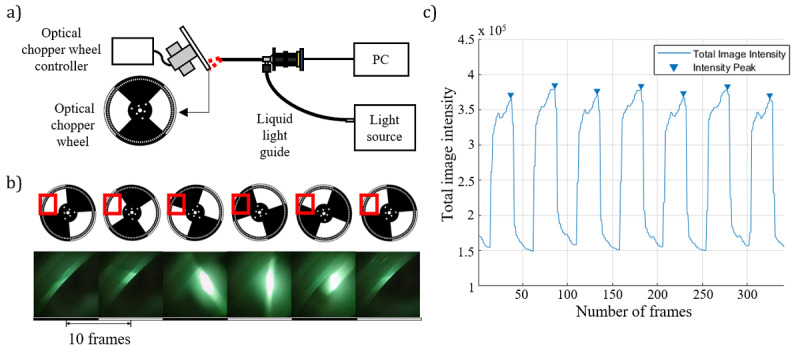
(A) Schematic of the system for high-speed imaging assessment. (B) Image series acquired over 50 frames. (C) A plot of the total image intensity by chopper wheel rotation. PC: personal computer.

A custom frame rate test was designed to confirm the video frame rate of the customized high-speed system. An optical chopper wheel was used to create a time-dependent moving sample of known frequency. Two quadrants of a 4-quadrant optical chopper wheel mask were optically blocked, and the other 2 quadrants were left transparent as shown schematically in Figure 4. While the light source provided illumination from the endoscope, high frame rate videos were acquired with the optical chopper wheel rotating at 10 Hz. The acquired high frame rate videos were analyzed by image processing in MATLAB, as light reflected from the chopper wheel was captured by the high-speed imaging system. The total intensity of light reflected from the 2 quadrants of the chopper wheel resulted in a series of image frames with a periodically modulated intensity signal that could be plotted frame by frame. The period of intensity modulation was calculated as the time interval between frames with peak intensities and was equal to half the rotational period of the optical chopper; this was measured to be 46.972 frames. Since the optical chopper was rotating at 10 Hz, the duration of a half-rotation was 0.05 seconds; therefore, the actual frame rate of the smartphone was 939.44 fps.

### Clinical Vocal Cord Vibration HSDI

**Figure 5:**
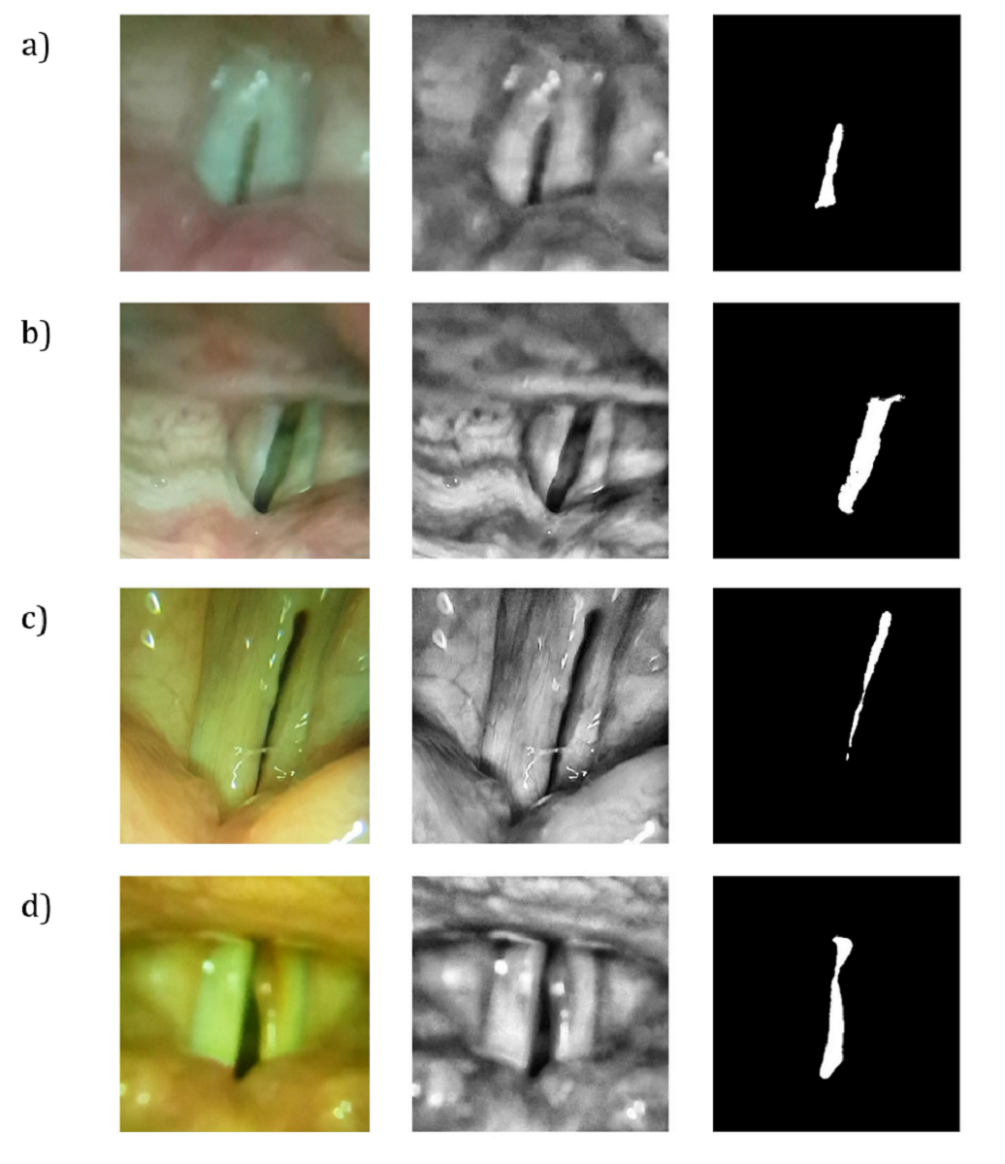
High-speed images of the glottis and their segmentation. Raw vocal cord image (left), preprocessed image for segmentation (center), and area of the normal vocal cord by image segmentation (right). (A) The glottis of a healthy volunteer. (B) Images taken from a patient with left vocal cord paralysis. (C) Vocal analysis from a patient with chronic laryngitis. (D) Images from a patient with a right vocal cord polyp.

**Figure 6:**
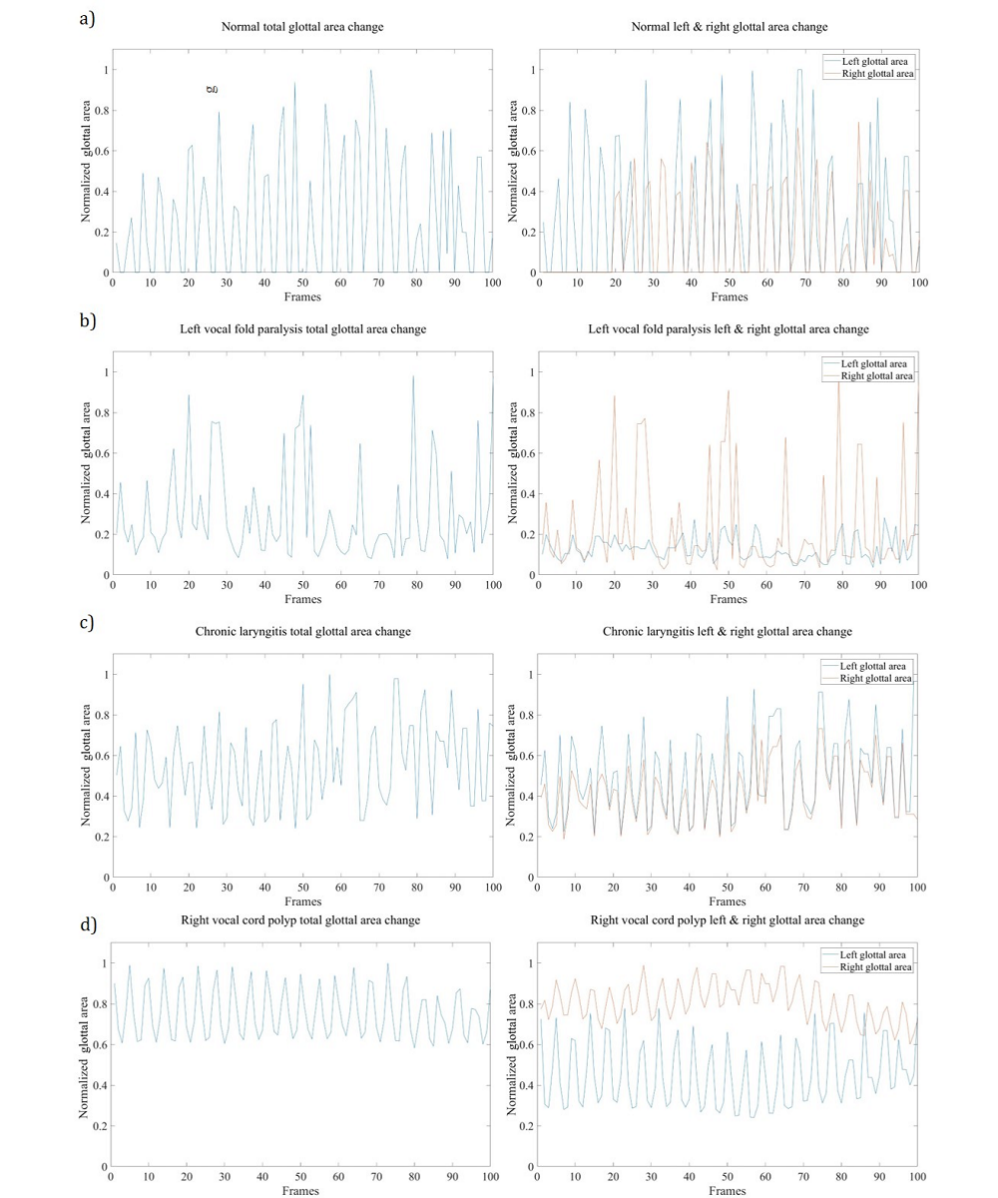
Plots of the normalized total glottal area (left) and a comparison of the normalized left and right glottal areas separated by the anatomical midline of the glottis over the course of a 0.4-second video at 940 fps (right) plotted for each patient: (A) normal healthy subject, (B) patient with left vocal cord paralysis, (C) patient with chronic laryngitis, and (D) patient with right vocal cord polyp.

The obtained HSDI vocal cord vibration videos were taken through a clinical diagnosis process. The results are presented in Figures 5 and 6. As depicted in Figure 5, the 940-fps HSDI video obtained from the clinical experiments was segmented into vocal cord area and glottal area for further analysis; this allowed us to obtain the parameters of the vocal cord with the largest contribution to clinical diagnosis. The parameters obtained from the vocal cord vibration HSDI data included the base vibration frequency of the vocal cord, the difference between the left glottal area and the right glottal area, and the total glottal area.

As shown in Figure 5, the data were obtained from the healthy subject, and patients with left vocal cord paralysis, chronic laryngitis, and right vocal cord polyp. The original RGB (red-green-blue) color image data were converted to grayscale, and the contrast was adjusted for glottal area segmentation using MATLAB. The seeded region growing method [30] was used to segment the glottal area from the image processed data. In addition, the anatomical midline of the vocal cord was drawn, after consultation with a clinician, to divide the left and right glottal areas.

The segmented glottal areas were analyzed in order to determine any differences between the data of the healthy subject and those of patients. From healthy subject data, normalized total glottal area changes are shown in Figure 6A. Using the fast Fourier transform, we were able to remove motion error and extract the base frequency of the healthy vocal cord. The fundamental frequency of the healthy subject, a 24-year-old female, was 224 Hz. The average fundamental frequency of adult females of this age is reported to be similar, at around 217 Hz [33]. Figure 6B shows the normalized total and lateral (left and right) glottal area changes in the vocal cords of a patient with left vocal cord paralysis. These data show that the amplitudes of the left vocal cord vibrations are significantly lower than those of the right vocal cord. The normalized total and lateral glottal areas of the patient with chronic laryngitis are shown in Figure 6C. Although the maximum amplitudes are similar to those of the healthy subject in both vocal cords, the minimum amplitude of each cycle differs from those of the healthy subject. The minimum amplitude value of each cycle in the healthy subject had almost zero area; this is because the vocal cord closes completely when the vocalizations are generated in a healthy subject. However, in the patient with chronic laryngitis, the minimum glottal area was larger than that in the healthy subject, because in chronic laryngitis the vocal cords do not close completely as sound is generated. Figure 6D shows the normalized total and lateral glottal areas for the vocal cords of a patient with a polyp on the right vocal cord. In this case, the vibrations of the right vocal cord were restricted by the polyp; therefore, the vibration amplitude of the right glottal area is less than that of the left glottal area. Similar to chronic laryngitis, the patient with the right vocal cord polyp had much larger minimum areas than the healthy subject; this phenomenon is caused by imperfect closure of the vocal cords due to the polyp.

## Discussion

### Principal Findings

Although many point-of-care devices have been developed based on smartphone systems, the full use of smartphone capabilities has been limited. Most smartphone-based point-of-care systems use chemical reactions of bodily fluids, including blood, saliva, sweat, and urine [18,34-36], and usage of smartphone functionalities are restricted to recognition of color changes or receiving electric signals generated by biochemical reactions. Most point-of-care devices are invasive, require bodily fluids, and their methods have limited applicability for noninvasive diagnostics. In these cases, the function of the camera is restricted to basic image acquisition [34]. Another way to integrate the smartphone into a point-of-care device would be by using the built-in functionality of a smartphone. Recently, consumer demand has resulted in rapid upgrades to embedded smartphone camera technology, resulting in unique functionality that can be incorporated into a special purpose imaging device.

Although commercialized HSDI devices enable unparalleled diagnoses, they also have several limitations. Their high cost and need for additional connection to video transmission processors like PCI-e (Peripheral Component Interconnect Express) frame grabbers make their application difficult for users in underdeveloped countries. In comparison, our smartphone-based HSDI system combines a printed endoscope-smartphone adapter and a low-cost commercial lens, demonstrating that even with low-cost components, it is possible to acquire high-speed digital images of vocal cord vibrations with existing clinical endoscopes.

Normally, images of vocal cord vibrations obtained by HSDI are postprocessed before being used in actual clinical practice. First, masking areas are drawn manually by the clinician, and the area is then plotted or processed for clinically relevant parameters. While this is traditionally performed by examining a series of individual frames superimposed on a laryngeal paralysis stroboscopic recording, or digital kymograms superimposed on wavefronts [37-39], modern computing makes it possible to extract glottal areas frame by frame using semiautomated image segmentation. As a result, the vocal cord areas and the glottal areas are divided and segmented as shown in Figure 5A. The clinical diagnostics obtained by the above process are the base cord and lateral phase difference of the vocal cord. The base cord is defined as the amount of change in the area of the entire glottal region, while the lateral phase difference is defined as the amount of change in the edge length of the left and right glottal areas [40].

The patient data obtained from our device made it possible to identify some well-known differences between patients with voice pathologies and the healthy subject. These results suggest that the smartphone-based HSDI equipment has the potential to be used to diagnose various voice diseases.

We first obtained clinical control data from a healthy female volunteer. The fundamental frequency appeared to be 224 Hz, which is similar to the average vocal cord fundamental frequency of adult females in this age range, which has been reported to be 217 Hz [33]. Although typical HSDI guidelines call for 7 to 10 frames per vibrational period, in this demonstration vocal cord period, amplitudes at normal speaking frequencies were successfully captured with 3- to 4-fold frame rates, demonstrating the suitability of this technology for clinical diagnostics.

Several clinically relevant features that differentiated the healthy control from the patients with voice pathologies were successfully observed. As shown in Figure 6, diagnosis of glottal pathologies can be easily obtained from plots of the vocal cord areas as calculated by segmentation of the glottal area in the images. In all patients, vocal impairments were shown by the limited amplitude of glottal vibration. In the patient with the one-sided vocal cord paralysis, the diagnosis presented as severely limited vibrational amplitude on one side, while chronic laryngitis presented with limited closure of the vocal cords. A polyp on one side of the focal cord displayed both limited closure and limited vibrational amplitude. Although our system does not achieve the high frame rate of clinical videokymography [37], the data analysis achieved from a 0.4 second clip is comparable in quality to that of videostroboscopy [38,41], capturing phase shifts of vibration, amplitude, vibrational asymmetry, and characteristic immobilization for diagnosis.

Commercially available HSDI systems, as shown in Table 1, have list prices of more than $10,000, and require installation of PCI cards to acquire an image series. Conversely, the smartphone-based HSDI system developed in this study can be configured for less than $500, which includes manufacturing of all 3D-printed parts and lens optics. The manufacturing costs of plastic parts could be further reduced by mass production. Furthermore, no additional connection interfaces are required to capture video, since the video interface used is included in the smartphone software. These calculations exclude the price of the smartphone, as smartphones are widely distributed in underserved markets. However, even if specific models of smartphone are required for smartphone-based HSDI systems, the price of a new smartphone is significantly less than that of a standard HSDI system. In addition, since smartphones can be connected to both Wi-Fi and mobile internet, users can easily transfer the captured video via wireless communication for analysis in remote regions. In developing countries, large hospitals may be significantly removed from the point of care, and this mobility can help to distribute medical service more equitably. Our system is simple and has intuitive maintenance requirements; therefore, it is easier to educate medical students in countries where medical schools are limited, although requirements for the cleaning of endoscopes complicate training and distribution.

**Table 1:** Comparison between a commercial high-speed digital imaging (HSDI) system and the smartphone-based HSDI system.

Characteristic	Commercial HSDI system (FASTCAM MC2, Photron)	Smartphone-based HSDI system
Frame rate	4000 fps	940 fps
Pixel count	512 × 512	1280 × 720
Extra connection interface	Gigabit Ethernet	USB 3.0 port
Price	>USD $10,000, excluding computer	<USD $400, excluding smartphone
Size	Camera: 35 mm × 35 mm × 34 mm Processor: 195 mm × 159 mm × 130 mm	Smartphone: 76 mm × 162 mm × 9 mm Adaptor: 115 mm × 120 mm × 80 mm
Weight	Camera: 100 g Processor: 5000 g Total : 5100 g	Smartphone: 201 g Adaptor: 295 g Total: 496 g

In addition, the smartphone-based HSDI system has a higher resolution than a traditional videostroboscope. Although the main focus of HSDI is a high frame rate, higher resolution enables the user to observe details that can assist with diagnosis. However, the advantages of mobility and price come with trade-offs in the form of device performance. Due to the limitations of the built-in smartphone functions, images can only be acquired at a maximum of 940 fps, which is considerably lower than that of the commercial HSDI system, which can achieve 4000 to 8000 fps. Typically, the fundamental vocal cord frequency for patients aged 17 to 25 years ranges from 115-132 Hz for males and 200-260 Hz for females [33]. Therefore, the smartphone-based system only captures 3 to 8 frames per vibration cycle, whereas 15 to 34 frames would be captured per vibration with the traditional HSDI. This frame rate is sufficiently high to detect the tendency of changes in the vocal cord area, but not sufficient to observe the details of each vibration cycle. This restriction may omit the detail necessary to diagnose certain conditions of the vocal cord. Therefore, additional filtering is required for frequency analysis.

Furthermore, the limitation on image capture duration to only 0.4 seconds at a time results in some pitfalls for clinical imaging, as it may be necessary for clinicians to repeat imaging in order to secure sufficient data for clinical evaluation. Although 0.4 seconds corresponds to 376 frames (47 vibrations), a clinician may have to take several HSDI videos from the smartphone to obtain sufficient data for analysis. Another drawback of the smartphone-based HSDI system developed in this study is that it requires a stronger endoscopic light source. As the charge-coupled device of smartphone cameras has lower photosensitivity than commercially available HSDI systems, a normal endoscope light source would not yield quality data. The stronger light source requires more power and can cause heat dissipation to become a consideration in the design of the device.

In the future, to overcome the aforementioned limitations, the development of more compact and optically shielded adaptors will be required. Emerging imaging solutions such as artificial intelligence–based image processing and deep learning could be applied to HSDI images of the vocal cord to overcome inadequate frame rates and acquisition times.

### Conclusions

Recent advances in smartphone technology, particularly in the domain of image sensors, have produced technology which rivals the performance of existing commercial high-speed cameras. In this study, we investigated whether the HSDI system currently used for vocal cord assessment can be replaced to some extent by using the high-speed video acquisition function from a smartphone camera. In validating the imaging capabilities of our smartphone-based high-speed endoscopy system against existing HSDI technology, our results demonstrate that it is possible to process clinical images for useful diagnostic reference values of healthy subjects. Furthermore, the possibility of disease diagnosis for actual patients was presented. Further development of smartphone-based HSDI technology is necessary to improve the distribution of health care capabilities.

## Abbreviations

CT: computed tomography
ELISA: enzyme-linked immunosorbent assay
FOV: field of view
HD: high definition
HSDI: high-speed digital imaging
PCI-e: Peripheral Component Interconnect Express
RGB: red-green-blue
SLM: selective laser melting

## References

[1] Rosen CA, Murry T (2000). Diagnostic laryngeal endoscopy. Otolaryngologic Clinics of North America.

[2] van Rossum PS, Goense L, Meziani J, Reitsma JB, Siersema PD, Vleggaar FP, van Vulpen M, Meijer GJ, Ruurda JP, van Hillegersberg R (2016). Endoscopic biopsy and EUS for the detection of pathologic complete response after neoadjuvant chemoradiotherapy in esophageal cancer: a systematic review and meta-analysis. Gastrointest Endosc.

[3] Davila RE, Rajan E, Adler D, Hirota WK, Jacobson BC, Leighton JA, Qureshi W, Zuckerman MJ, Fanelli R, Hambrick D, Baron TH, Faigel DO (2005). ASGE guideline: the role of endoscopy in the diagnosis, staging, and management of colorectal cancer. Gastrointestinal Endoscopy.

[4] Schlegel P, Kunduk M, Stingl M, Semmler M, Döllinger Michael, Bohr C, Schützenberger Anne (2019). Influence of spatial camera resolution in high-speed videoendoscopy on laryngeal parameters. PLoS One.

[5] Deliyski DD, Petrushev PP, Bonilha HS, Gerlach TT, Martin-Harris B, Hillman RE (2008). Clinical implementation of laryngeal high-speed videoendoscopy: challenges and evolution. Folia Phoniatr Logop.

[6] Poburka BJ, Patel RR, Bless DM (2017). Voice-Vibratory Assessment With Laryngeal Imaging (VALI) Form: Reliability of Rating Stroboscopy and High-speed Videoendoscopy. J Voice.

[7] Mehta DD, Deliyski DD, Quatieri TF, Hillman RE (2011). Automated Measurement of Vocal Fold Vibratory Asymmetry From High-Speed Videoendoscopy Recordings. J Speech Lang Hear Res.

[8] Bohr C, Kraeck A, Eysholdt U, Ziethe A, Döllinger Michael (2013). Quantitative analysis of organic vocal fold pathologies in females by high-speed endoscopy. Laryngoscope.

[9] Bless D, Hirano M, Feder R J (1987). Videostroboscopic evaluation of the larynx. Ear Nose Throat J.

[10] Hirose H (1988). High-speed digital imaging of vocal fold vibration. Acta Otolaryngol Suppl.

[11] Schutte HK, Svec J G, Sram F (1998). First results of clinical application of videokymography. Laryngoscope.

[12] Schade G, Müller F (2005). [High speed glottographic diagnostics in laryngology]. HNO.

[13] Echternach M, Sataloff RT (2017). High-Speed Digital Imaging. Neurolaryngology.

[14] Guzman M, Laukkanen A, Traser L, Geneid A, Richter B, Muñoz Daniel, Echternach M (2017). The influence of water resistance therapy on vocal fold vibration: a high-speed digital imaging study. Logoped Phoniatr Vocol.

[15] Lohscheller J, Dollinger M, Schuster M, Schwarz R, Eysholdt U, Hoppe U (2004). Quantitative Investigation of the Vibration Pattern of the Substitute Voice Generator. IEEE Trans Biomed Eng.

[16] Yamauchi A, Yokonishi H, Imagawa H, Sakakibara K, Nito T, Tayama N, Yamasoba T (2016). Quantification of Vocal Fold Vibration in Various Laryngeal Disorders Using High-Speed Digital Imaging. J Voice.

[17] Nwokediuko SC (2011). Challenges of Gastrointestinal Endoscopy in Resource-Poor Countries. Gastrointestinal Endoscopy.

[18] St John Andrew, Price C (2014). Existing and Emerging Technologies for Point-of-Care Testing. Clin Biochem Rev.

[19] del Rosario M, Redmond S, Lovell N (2015). Tracking the Evolution of Smartphone Sensing for Monitoring Human Movement. Sensors (Basel).

[20] Soraya G, Nguyen T, Abeyrathne C, Huynh D, Chan J, Nguyen P, Nasr B, Chana G, Kwan P, Skafidas E (2017). A Label-Free, Quantitative Fecal Hemoglobin Detection Platform for Colorectal Cancer Screening. Biosensors (Basel).

[21] Guo J (2017). Smartphone-Powered Electrochemical Dongle for Point-of-Care Monitoring of Blood β-Ketone. Anal Chem.

[22] Breslauer DN, Maamari RN, Switz NA, Lam WA, Fletcher DA (2009). Mobile phone based clinical microscopy for global health applications. PLoS One.

[23] Mistry N, Coulson C, George A (2017). endoscope-i: an innovation in mobile endoscopic technology transforming the delivery of patient care in otolaryngology. Expert Rev Med Devices.

[24] Bae JK, Vavilin A, You JS, Kim H, Ryu SY, Jang JH, Jung W (2017). Smartphone-Based Endoscope System for Advanced Point-of-Care Diagnostics: Feasibility Study. JMIR Mhealth Uhealth.

[25] Ha JHI, Sagili SR (2019). Smartphone adaptor use for nasal endoscopy. Eye (Lond).

[26] Quimby AE, Kohlert S, Caulley L, Bromwich M (2018). Smartphone adapters for flexible Nasolaryngoscopy: a systematic review. J Otolaryngol Head Neck Surg.

[27] Moon Y, Oh J, Hyun J, Kim Y, Choi J, Namgoong J, Kim J.K (2020). Cost-Effective Smartphone-Based Articulable Endoscope Systems for Developing Countries: Instrument Validation Study. JMIR Mhealth Uhealth.

[28] Agu E, Pedersen P, Strong D, Tulu B, He Q, Wang L, Li Y (2013). The smartphone as a medical device: Assessing enablers, benefits and challenges.

[29] (2018). The Official Samsung Galaxy Site: Specifications. Samsung.

[30] Adams R, Bischof L (1994). Seeded region growing. IEEE Trans. Pattern Anal. Machine Intell.

[31] Schlegel P, Kniesburges S, Dürr Stephan, Schützenberger Anne, Döllinger Michael (2020). Machine learning based identification of relevant parameters for functional voice disorders derived from endoscopic high-speed recordings. Sci Rep.

[32] Moses FM, Lee JS (2004). Current GI endoscope disinfection and QA practices. Dig Dis Sci.

[33] Huss PJ (1983). Vocal Pitch Range and Habitual Pitch Level: The Study of Normal College Age Speakers. Master's Theses.Western Michigan University.

[34] Xu X, Akay A, Wei H, Wang S, Pingguan-Murphy B, Erlandsson B, Li X, Lee W, Hu J, Wang L, Xu F (2015). Advances in Smartphone-Based Point-of-Care Diagnostics. Proc. IEEE.

[35] Roda A, Michelini E, Zangheri M, Di Fusco M, Calabria D, Simoni P (2016). Smartphone-based biosensors: A critical review and perspectives. TrAC Trends in Analytical Chemistry.

[36] Liu J, Geng Z, Fan Z, Liu J, Chen H (2019). Point-of-care testing based on smartphone: The current state-of-the-art (2017-2018). Biosens Bioelectron.

[37] Eysholdt U, Tigges M, Wittenberg T, Pröschel U (1996). Direct evaluation of high-speed recordings of vocal fold vibrations. Folia Phoniatr Logop.

[38] Sercarz JA, Berke GS, Ming Y, Gerratt B R, Natividad M (1992). Videostroboscopy of human vocal fold paralysis. Ann Otol Rhinol Laryngol.

[39] Wittenberg T, Tigges M, Mergell P, Eysholdt U (2000). Functional imaging of vocal fold vibration: Digital multislice high-speed kymography. Journal of Voice.

[40] Sulter AM, Schutte HK, Miller DG (1996). Standardized laryngeal videostroboscopic rating: Differences between untrained and trained male and female subjects, and effects of varying sound intensity, fundamental frequency, and age. Journal of Voice.

[41] Banjara H, Mungutwar V, Singh D, Gupta A, Singh S (2012). Demographic and videostroboscopic assessment of vocal pathologies. Indian J Otolaryngol Head Neck Surg.

